# miRNA-Mediated Signaling Networks in Non-Small Cell Lung Cancer: Linking Tumor Progression to Sarcopenia

**DOI:** 10.3390/ijms27114703

**Published:** 2026-05-23

**Authors:** Swati Goswami, Pooja Gulhane, Shailza Singh

**Affiliations:** 1 Systems Medicine Laboratory, BRIC—National Centre for Cell Science, National Centre for Cell Science Complex, SPPU Campus, Ganeshkhind, Pune 411007, India; 2 Regional Centre for Biotechnology, Faridabad 121001, India

**Keywords:** cancer, miRNA, NSCLC, FOXO1, TME, sarcopenia

## Abstract

Non-small cell lung cancer (NSCLC) remains a major cause of cancer-related mortality, with poor survival outcomes despite advances in surgery, chemotherapy, targeted therapy, and immunotherapy. The tumor microenvironment (TME) plays a central role in sustaining tumor growth, immune evasion, and systemic metabolic dysfunction. In this study, we performed an integrative analysis of differentially expressed microRNAs (miRNAs) to uncover their contributions to dysregulated signaling networks in NSCLC. hsa-miR-486-5p was identified as a prominent differentially expressed candidate miRNA. Using mathematical modeling and regression-based reduction, we identified Forkhead Box O1 (FOXO1) and Unc-51 like Autophagy Activating Kinase 2 (ULK2) as critical regulatory nodes that integrate oncogenic signaling with cellular homeostasis. Aberrant expression of hsa-miR-486-5p was found to modulate pathways including PI3K/AKT/mTOR, NF-κB, and JAK-STAT3, thereby promoting tumor progression and secretion of inflammatory cytokines. These cytokines, viz., IL-6, TNF-α, and IL-1β, activate muscle-specific protein degradation pathways through E3 ubiquitin ligases TRIM63 and FBXO32, linking NSCLC progression to cancer-associated sarcopenia. Quasipotential landscape analysis further revealed dynamic phenotypic transitions between stable and unstable states, highlighting the adaptability of tumor–host interactions. Collectively, our findings demonstrate that miRNA-mediated regulatory networks not only drive NSCLC progression and inflammation but also contribute to systemic muscle wasting. These insights emphasize the need for novel therapeutic strategies, including RNA-based interventions, to overcome resistance, improve survival, and address the metabolic complications associated with NSCLC.

## 1. Introduction

Among the non-communicable diseases, cancer remains one of the leading causes of mortality all over the globe. The estimated GLOBOCAN 2022 data depicted approximately 20 million new cases of cancer and nearly 10 million deaths due to cancer worldwide. With nearly 2.48 million new cases and 1.8 million deaths reported in 2022, lung cancer has become the most commonly diagnosed cancer around the world, making it a serious public health concern. For both sexes combined, lung cancer alone accounts for nearly 18% of the total deaths caused by cancer every year. Incidence of lung cancer is more prevalent among males, exhibiting ≈1.57 million cases as compared with females, accounting for ≈0.91 million cases [1]. The latest Indian data from the National Cancer Registry Programme (NCRP), reported for the years 2012–16, classified lung cancer as the leading type of cancer significantly prevalent in metropolitan areas [2,3]. Lung cancer can be classified as small cell lung cancer (SCLC) and non-small cell lung cancer (NSCLC), accounting for nearly 15% and 85% of total lung cancer, respectively. NSCLC accounts for major cases of lung cancer and is broadly classified as lung adenocarcinoma, squamous cell carcinoma, and large cell carcinoma [4,5,6]. Based upon an Indian Council of Medical Research (ICMR) study of different Indian populations, adenocarcinoma showed persistent prevalence surpassing squamous cell carcinoma and large cell carcinoma in both sexes [3].

NSCLC has also been classified based on the type of mutation it harbors, such as epidermal growth factor receptor (EGFR), Kirsten rat sarcoma virus (K-RAS), and anaplastic lymphoma kinase (ALK), which has led to the development of precision medicine for NSCLC treatment [7,8]. Non-small cell lung cancer (NSCLC) treatment involves a multimodal approach, including surgery, radiation, chemotherapy, targeted therapy, and immunotherapy [9,10]. However, resistance and relapse remain major challenges, and emerging RNA-based therapies represent a promising frontier, offering novel mechanisms to target oncogenic drivers and immune evasion, potentially complementing existing treatments and improving long-term survival [11,12].

Different RNA molecules, such as antisense oligonucleotides (ASOs), small interfering RNAs (siRNAs), and microRNAs (miRNAs) have already been investigated for their therapeutic role in different disease models, including cancer [13,14]. MiRNA-based therapeutics have shown promising outcomes in various cancers, acting as both oncogenes and tumor suppressors [15]. MiRNA is one such small non-coding RNA—nearly 22 nucleotides in length—regulating the post-transcriptional expression of different genes. MiRNA binds to target mRNA and results in silencing and suppression of the target gene. MiR-21 has been reported to target tumor suppressor genes such as phosphatase and tensin homolog (PTEN), programmed cell death 4 (PDCD4), and tropomyosin 1 (TPM1) in lung, gastric, brain, and breast cancers [16]. Likewise, miR-221 has been shown to inhibit P27 and P57, the cell cycle inhibitors, and thereby promote tumor progression in colon carcinoma, renal carcinoma, and hepatocellular carcinoma [17]. Various tumor suppressive miRNAs have also been reported, such as miR-143 and miR-145, having reduced expression in lung, breast, bladder, and colorectal cancers [18,19,20]. In order to ensure the stability of miRNA in targeted therapeutics, an efficient delivery system is required. Nanoparticle-based carriers and exosomes have been intensively studied for this purpose [21]. Exosomes are tiny (30–150 nm) extracellular vesicles secreted by almost every cell of the body, including cancer cells. Exosomes consist of DNA, RNA, proteins, and other biomolecules. These exosomes are responsible for the shuttling of miRNAs and other molecular cues between cancer cells and the neighboring cells. In the tumor microenvironment (TME), these exosomes mediate communication to promote tumor progression, outpace the immune response, resistance against therapies, metastasis, and angiogenesis [22].

TME supports the activation of various signaling pathways, which are tumorigenic, inflammation-causing, and stress-inducing, to promote the growth and survival of tumor cells. The phosphoinositide-3-kinase/protein kinase B/mechanistic target of rapamycin (PI3K-AKT-mTOR) axis is central to the overall cancer signaling pathway, which activates downstream oncogenic signal cascades [23,24]. Among solid tumors, another prominent signaling molecule is hypoxia-inducible factor-1-α (HIF-1 α). The enhanced glycolysis, angiogenesis, and improper vascularization enable cancer cells to adapt to hypoxic conditions. Hypoxia results in the formation of reactive oxygen species (ROS), which in turn promotes tumor progression [25,26]. The inflammator33y signaling inside TME is activated due to the nuclear factor kappa-light chain enhancer of activated B cells (NF-kB) and Janus kinase–signal transducer and activator of transcription 3 (JAK-STAT3). These activate the secretion of cytokines such as interleukin-6 (IL-6), interleukin-1β (IL-1β), and tumor necrosis factor-α (TNF-α) from the tumor cells and associated stromal cells for the progression of cancer and to support the immunosuppressive environment [27]. These cytokines also lead to activation of the protein degradation pathway in muscle cells, leading to muscle atrophy [25]. Together, these tumor-promoting and inflammatory signals within the TME not only sustain cancer cell survival but also impose systemic stress on surrounding tissues. This cellular stress response converges on transcriptional regulators, such as forkhead box O (FOXO) proteins, which act as critical mediators linking oncogenic signaling, inflammation, and metabolic imbalance to both tumor progression and muscle wasting [28]. FOXO-1 has also been reported to activate atrogin-1 (FBXO32), which is a muscle-specific E3-ubiquitin ligase, and MuRF-1 (TRIM63), both degenerate the myofibril proteins, triggering cancer-associated sarcopenia [29,30,31]. Autophagy has a context-dependent role in TME. At an early stage, it helps in tumor suppression, and in later stages, it promotes tumor progression [32,33].

In this study, we employed integrated sequence analysis to identify miRNAs with significant differential expression. Using a systems biology framework combined with network analysis, we uncovered disruptions in cancer-associated signaling pathways that govern tumor growth, survival, and systemic metabolic alterations contributing to sarcopenia. Mathematical modeling of cancer and muscle cell signaling has highlighted the pivotal role of FOXO-1 in mediating crosstalk between these cell types. While numerous miRNAs have been reported to regulate diverse genes, the regulation of FOXO-1 and ULK2 by microRNAs in non-small cell lung cancer (NSCLC) remains unexplored. Our findings emphasize the importance of hsa-miR-486-5p in modulating FOXO-1, thereby shedding light on its functions in both tumor progression and muscle wasting.

## 2. Results

### 2.1. Cross-Platform Differential Expression Analysis Revealed Crucial miRNAs in NSCLC

**Figure 1:**
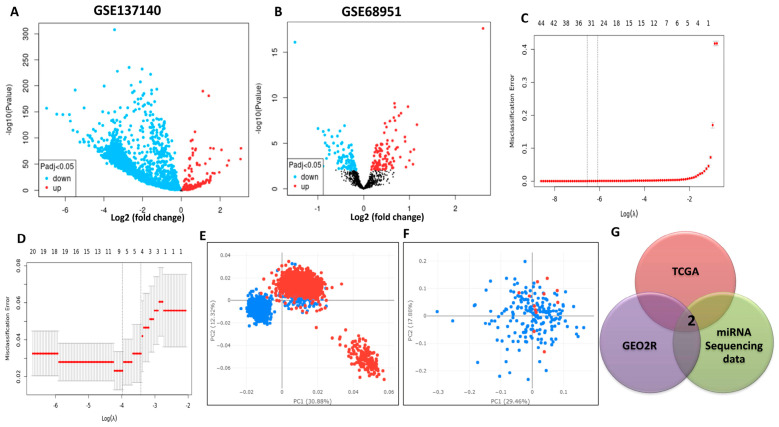
Integrated differential expression analysis of microRNAs in NSCLC. (**A**,**B**) Volcano plots of differentially expressed microRNAs in GSE137140 and GSE68951, showing log_2_ fold change versus −log_10_(*p* value); significantly upregulated (red) and downregulated (blue) microRNAs are highlighted (adj. *p* < 0.05). (**C**,**D**) LASSO regression plots indicating statistically significant microRNAs after adjustment for GSE137140 and GSE68951, respectively. (**E**,**F**) Principal component analysis (PCA) showing separation of NSCLC and control samples based on microRNA expression profiles. (**G**) Venn diagram showing overlap of significantly dysregulated microRNAs across TCGA, GEO2R, and small RNA sequencing datasets.

An integrative screening of putative miRNAs was performed using TCGA (https://portal.gdc.cancer.gov/projects/tcga-luad Accessed: 12 October 2025), GEO2R (https://www.ncbi.nlm.nih.gov/geo/geo2r/ Accessed: 15 October 2025), small RNA sequencing of NSCLC cells, and CancerMIRNome (http://bioinfo.jialab-ucr.org/CancerMIRNome Accessed: 27 October 2025). Using the TCGABiolinks package in R (version 4.5.1), miRNA sequence data for LUAD was retrieved and was stored in the form of a matrix using the SummarizedExperiment package in R. DESeq2 was employed to screen for DE miRNAs, and a total of 310 significant miRNAs were identified based upon the statistical considerations of logFC value ≥ 1 showing at least a 2-fold upregulation and downregulation along with an adjusted *p*-value less than 0.05. The normalization criteria for the considered TCGA-LUAD was the DESeq2 size factor normalization method (Supplementary File S1). Similarly, GEO2R and CancerMIRNome were also used to find differentially expressed miRNAs in GSE137140 and GSE68951 (Supplementary Files S2 and S3, respectively). Using GEO2R, the groups (tumor and control) were defined, and the top 500 differentially expressed miRNAs were obtained using R (Limma package), represented by a volcano plot. Downregulated miRNAs are shown as blue dots, and upregulated miRNAs are shown as red dots. Asymmetric distribution of miRNAs was found in the case of GSE137140 (Figure 1A), while nearly symmetric distribution of miRNAs was seen in the case of the GSE68951 dataset (Figure 1B). Small RNA sequencing of two NSCLC cell lines, H1975 and A549, when compared with control Beas-2b, revealed that, among the top 500 DE miRNAs from both the cell lines, 56.4% were found in common (Supplementary Files S4 and S5). Normalization of datasets was done using DESeq2 for small RNA sequencing. We also performed the least absolute shrinkage and selection operator (LASSO) regression analysis using CancerMIRNome for the two datasets, which revealed stable and minimal miRNA candidates by performing variable selection and regularization. In the dataset GSE137140, a higher proportion of miRNAs was retained in the model at lower λ values, which resulted in reduced penalization and a more complex model. The number of selected miRNAs decreased as λ increased because regression coefficients gradually declined towards zero. Over a range of λ values, the cross-validated misclassification error remains low, suggesting that a small selection of miRNAs could provide the best classification performance (Figure 1C). For dataset GSE68951, the red points represent the mean misclassification error across cross-validation folds, while the error bars indicate variability in model performance. Considering a restricted standard error, the lowest error was found close to the ideal λ range, indicating robust performance in classification throughout various data segments. Misclassification error significantly increased as λ exceeded this ideal range, indicating under-fitting caused by excessive penalization (Figure 1D).

**Figure 2:**
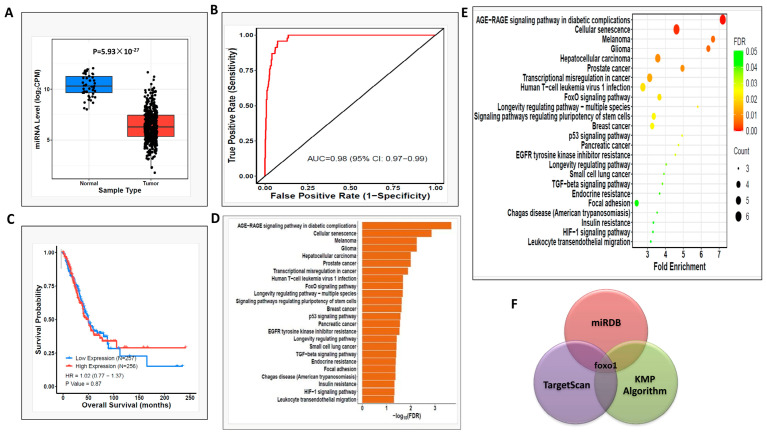
Expression, prognostic significance, and functional analysis of hsa-miR-486-5p in NSCLC. (**A**) Box plot showing differential expression of hsa-miR-486-5p between normal and tumor samples. (**B**) ROC curve demonstrating diagnostic performance of hsa-miR-486-5p. (**C**) Kaplan–Meier survival analysis comparing overall survival between high and low hsa-miR-486-5p expression groups. (**D**) Bar plot of significantly enriched pathways for predicted target genes of hsa-miR-486-5p. (**E**) Bubble plot showing pathway enrichment with fold enrichment and FDR values. (**F**) Venn diagram illustrating the overlap of target genes predicted by miRDB, TargetScan, and KMP-based algorithms, identifying FOXO1 as a common target.

Furthermore, we generated principal component (PC) plots for both datasets. In the case of GSE137140, cancer and control samples showed considerable overlap with minor variations, where PC1 explained 29.46% of the variance and PC2 accounted for 17.88% (Figure 2A). For GSE68951, the distribution revealed PC1 contributing 30.88% of the variance and PC2 explaining 12.32% (Figure 1B). Furthermore, analysis of overlapping miRNAs between the two datasets identified a 13.3% shared subset, highlighting a degree of commonality in miRNA expression profiles across these studies.

On comparison of DE miRNAs based on logFC value and adjusted *p*-value obtained from TCGA, GSE datasets, and small RNA sequencing (A549 and H1975), only two statistically significant microRNAs were found, namely, hsa-miR-486-5p and hsa-miR-615-5p (Figure 1G). hsa-miR-486-5p was found to be significantly downregulated in A549 and H1975 sequencing data with log-FC value (−4.9155 and −7.50, resp.) and *p*-values nearly zero.

### 2.2. Expression of hsa-miR-486-5p in NSCLC and Altered Functional Pathways

Box plot analysis of hsa-miR-486-5p using the CancerMIRNome platform for normal and lung cancer was performed. The blue box represents the normal sample, the red box represents the tumor sample, and the black dots represent the number of samples considered for analysis. A significant downregulation of miRNA was seen in the case of tumor samples (Figure 2A). A receiver operative curve (ROC) plot of sensitivity vs. specificity was obtained, where the red line represents the ROC curve and the black line represents random classification. Area under the curve (AUC) is 0.98, depicting highly significant discriminative ability between normal and lung cancer samples. The 95% confidence interval is between 0.97 and 0.99, which shows that the data are robust and reliable (Figure 2B). The Kaplan–Meier survival curve demonstrated the overall survival with hsa-miR-486-5p low and high expression (Figure 2C). Pathway enrichment analysis was performed using CancerMIRNome, and a number of cancer and metabolism-related signaling pathways were found to be altered. The most enriched pathway was found to be the AGE-RAGE signaling pathway in diabetic complications, followed by cellular senescence, explaining the role of miRNA in oxidative stress, ageing, and disease progression. Several cancer pathways, such as melanoma, glioma, hepatocellular carcinoma, prostate cancer, breast cancer, and small cell lung cancer, were found to be altered, pointing towards the relevance of hsa-miR-486-5p in cancer occurrence. Cancer regulatory pathways mediated by FOXO, p53, HIF-1α, TGF-β, and EGFR-tyrosine kinase inhibitor were found to be altered, proposing their role in cancer progression, therapy resistance, and hypoxia (Figure 2D,E). Enrichment of all of these pathways suggests that dysregulation in this miRNA has an effect on tumor progression, metabolic reprogramming, and resistance against therapies. Further, for target prediction, an integrative approach using three different methods for target prediction through miRDB, TargetScan, and the KMP algorithm was utilized, and a common target was obtained (Figure 2F).

### 2.3. Pathway and miRNA-Mediated Dysregulation in Lung Adenocarcinoma

**Figure 3:**
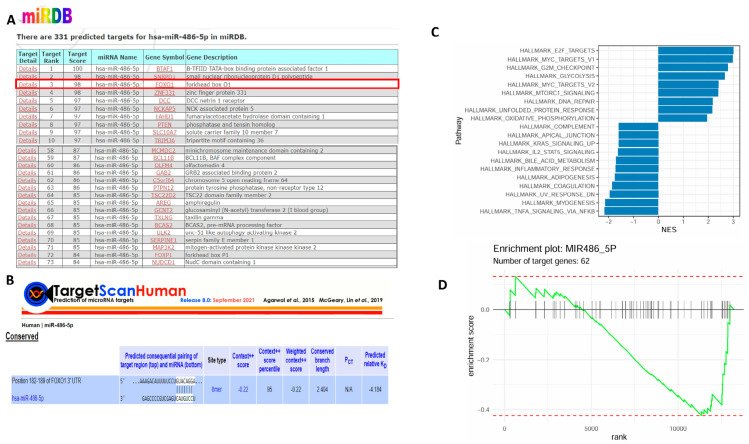
Target prediction and pathway enrichment analysis of hsa-miR-486-5p. (**A**) miRDB output showing predicted target genes of hsa-miR-486-5p, with FOXO1 highlighted as a high-confidence target. (**B**) TargetScan analysis indicating conserved binding sites of hsa-miR-486-5p within the 3′UTR of FOXO1. (**C**) Hallmark pathway enrichment analysis of hsa-miR-486-5p target genes showing positively and negatively enriched biological processes based on normalized enrichment score (NES). (**D**) GSEA enrichment plot for the MIR486_5P gene set, illustrating the distribution of target genes across the ranked gene list.

Gene set enrichment analysis of LUAD in comparison with the normal lung tissue of hallmark-associated pathways revealed a strong enrichment of proliferative and oncogenic signaling. Various pathways with both positive and negative enrichment scores were obtained. Top positive enriched targets were E2F targets, cell cycle G2M checkpoints, glycolysis, mTORC signaling, DNA repair, and unfolded protein response. The top negatively enriched pathways obtained were TNF-α signaling, myogenesis, UV response, coagulation, IL2-STAT signaling, and KRAS signaling (Figure 3C).

For the investigation of post-transcriptional regulatory mechanisms behind these pathway alterations, we performed miRNA-target-based GSEA [34]. Comparing target genes (TargetScan) with the ranked file (MsigDB) resulted in 62 common targets of hsa-miR-486-5p contributing to the leading-edge subset genes, including PTEN, FOXO1, PIK3R1, IGF1, and CDK4, involved in cell cycle regulation, growth signaling, and metabolic pathways, suggesting a notable link between miR-486-5p and the proliferative phenotype in LUAD. Enrichment plot showed downregulation of various genes related to lung differentiation and immune response was observed in NSCLC LUAD samples (Figure 3D), which again depicted the contribution of miR-486-5p to NSCLC progression by modulating the signaling network associated with cell cycle activation and metabolic reprogramming.

### 2.4. Target Prediction for miRNA

For the prediction of putative miRNA targets, different methods were used. At least three methods were used to select the significant target. MiRDB, TargetScan, and the KMP algorithm together revealed that FOXO1 can be a key target of hsa-miR-486-5p. Similarly, ULK2 has been confirmed using miRDB, RNAhybrid, and the KMP algorithm (Figure 3A). The target scores for FOXO1 and ULK2 in miRDB were 98 and 85, respectively. TargetScan with cumulative context++ score, and the context++ score percentile with −0.22 and 95, respectively, revealed that FOXO1 can be an important target of the curated miRNA (Figure 3B). This study relies on in silico target prediction and structural modeling of the Argonaute protein–microRNA–messenger RNA complex. While these approaches suggest that the predicted interactions are structurally feasible, they do not provide direct evidence of functional gene regulation.

### 2.5. Structural Analysis of the Argonaute–microRNA–mRNA Complex

**Figure 4:**
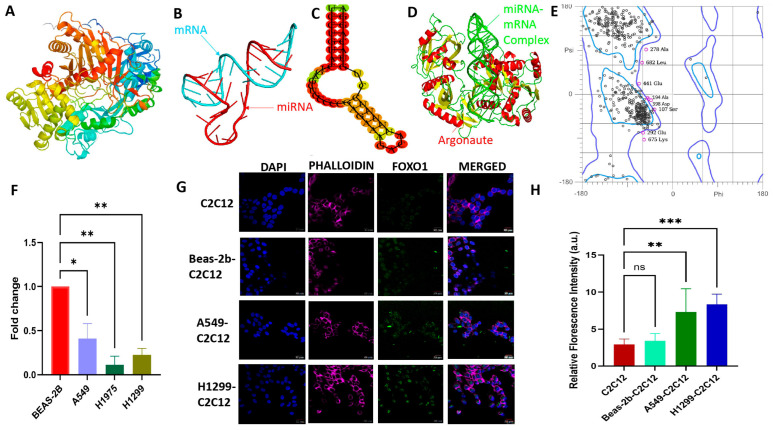
Structural modeling, validation of miRNA–mRNA interaction, and miRNA and FOXO-1 expression analysis in linking NSCLC with muscle wasting. (**A**) 3D structure of Argonaute protein (PDB ID: 3F73). (**B**) Predicted binding between miRNA (red) and target mRNA (cyan). (**C**) Secondary structure of the miRNA. (**D**) Tertiary structure of the miRNA–mRNA complex bound to Argonaute (green: miRNA–mRNA; red/yellow: Argonaute). (**E**) Ramachandran plot showing residue geometry validation of Argonaute structure. (**F**) Relative fold-change expression of target genes in different cell lines. (**G**) Beas-2b, A549, and H1299 cell lines were cultured with C2C12, where blue represents stained nucleus with DAPI, magenta represents stained cytoskeletal elements with phalloidin dye, and an antibody-mediated immunofluorescence against FOXO-1 is represented in green. (**H**) Mean fluorescence intensity was measured by quantifying the confocal results, and the relative fluorescence intensity graph showed significantly upregulated FOXO-1 expression in muscle cells co-cultured with A549 and H1299 cell lines (* *p* < 0.1, ** *p* < 0.01, *** *p* < 0.001, ns: non-significant).

Argonaute protein structure was obtained from RCSB PDB (Figure 4A), and the secondary structure of the miRNA–mRNA complex was generated using RNAfold (Figure 4C). For the tertiary structure of the complex, RNAComposer (https://rnacomposer.cs.put.poznan.pl/ Accessed: 23 December 2025) was utilized (Figure 4B). Ten distinct docking models of the microRNA–mRNA complex with Argonaute protein (PDB ID: 3F73) using GRAMM were generated (Figure 4D). To evaluate the thermodynamic stability of each model, PDBePISA was used. Among these, one complex exhibited a highly negative Gibbs free energy of binding (ΔG) of −56.7 kcal/mol, with the lowest ΔG *p*-value 0.250, indicating a highly stable protein–RNA interaction. This model was therefore selected as the most energetically favourable and biologically relevant complex for further structural analysis. Using a Ramachandran plot, the structural validation of the selected Argonaute–microRNA–mRNA complex was performed. The analysis depicted that the majority of amino acid residues were within the most favored and allowed regions (shown in black circles), with only a minimal number of residues falling in disallowed regions (Figure 4E). This distribution indicates acceptable stereochemical quality of the protein structure after docking and supports the reliability of the predicted complex. The modeled Argonaute–miRNA–mRNA complex demonstrates that the predicted binding site of hsa-miR-486-5p on *FOXO1* mRNA is structurally compatible. The seed region of the miRNA aligns with the complementary sequence in the target mRNA, supporting the possibility of post-transcriptional regulation. However, this structural analysis alone does not confirm functional repression and should be interpreted along with experimental data which demonstrate changes in FOXO1 expression.

### 2.6. Mathematical Modeling

**Figure 5:**
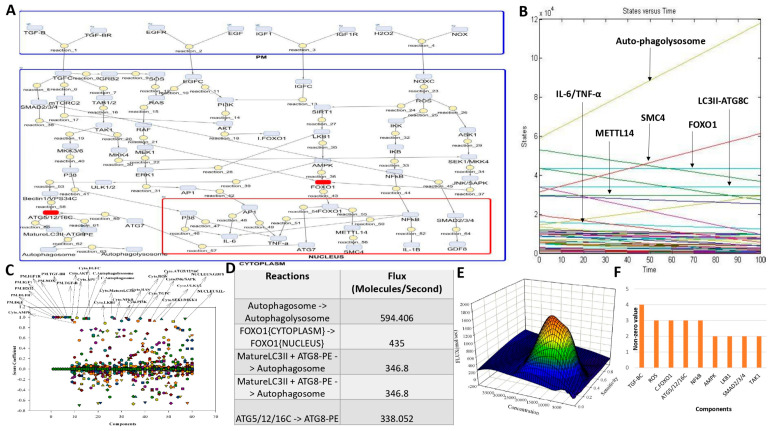
System level modeling of FOXO1-centered signaling and metabolic reprogramming in NSCLC. (**A**) Mathematical model depicting crosstalk between oncogenic, inflammatory, metabolic, and autophagy-related signaling pathways, highlighting FOXO1 as a central regulatory node. (**B**) Time-course simulation showing dynamic changes in key molecular states, including IL-6/TNF-α signaling, FOXO1 activity, and autophagolysosome formation. (**C**) PCA plot illustrating key regulatory molecules represented in dots across the modeled network. (**D**) Table summarizing flux values of key reactions involved in autophagy and FOXO1 translocation. (**E**) Three-dimensional quasipotential landscape showing system behavior across varying parameter concentrations. (**F**) Crosstalk point identifying FOXO1-associated components as major contributors to system variance.

Based on the pathway analysis, a mathematical model was reconstructed to investigate intracellular signaling dynamics in NSCLC. Implemented in MATLAB v7.11.1.866, the model incorporated three compartments representing the cell membrane, cytoplasm, and nucleus to simulate cellular conditions, which consists of 60 components, 64 reactions, and 126 kinetic laws (Figure 5A). Simulation outputs depicted key regulatory molecules, namely, autophagolysosome, IL6/TNF α, METTL14, SMC4, FOXO1, and the LC3II–ATG8 complex using distinct color codes (Figure 5B). Principal component analysis (PCA) identified critical interacting molecules with roles in cancer progression, epithelial–mesenchymal transition (EMT), autophagy, and cytokine secretion, each contributing to modulation of the tumor microenvironment (Figure 5C). The reactions and parameters involved are given in Supplementary Files S6, S7 and S10. Flux balance analysis further revealed high-flux reactions, including the conversion of autophagosomes to phagolysosomes, nuclear translocation of FOXO1, maturation of LC3-II for autophagosome membrane formation, and activation of ATG8-PE mediated by the ATG5/12/16 complex (Figure 5D). Model reduction further yielded a hat-shaped quasipotential landscape (Figure 5E), reflecting the dynamic stability states of the system and emphasizing the interconnected regulatory architecture that governs tumor progression and cellular adaptation. Crosstalks identified in the cancer cell mathematical model encompassed key signaling mediators, including TGF-β, ROS, FOXO1, the ATG5/12/16 complex, NFκB, AMPK, LKB1, SMAD2/3/4, and TAK1 (Figure 5F).

**Figure 6:**
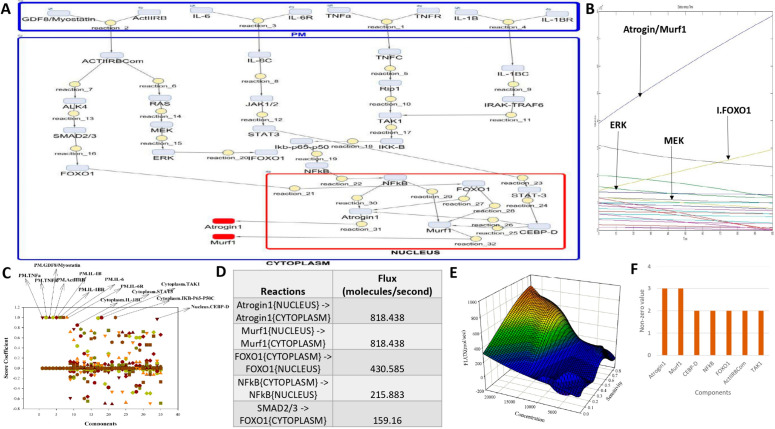
Simulation and analysis of the inflammation-induced signaling network in NSCLC-induced sarcopenia. (**A**) Network model depicting interactions among cytokines, receptors, and downstream effectors in the plasma membrane (PM), cytoplasm, and nucleus; key regulators Atrogin1 and Murf1 are highlighted in red. (**B**) Dynamic simulation of protein activity over time, showing trends for Atrogin1/Murf1, FOXO1, MEK, and ERK. (**C**) PCA plot of regulatory components across the network. (**D**) Calculated flux values (molecules/second) for key reactions. (**E**) Quasipotential landscape showing stability of components in the reconstructed model, (**F**) Crosstalk point for depiction of major network components.

To elucidate the mechanisms of cancer-associated muscle atrophy, an additional mathematical model was developed to analyze how tumor-derived molecules and cytokines drive the activation of atrophy-related genes. In total, three compartments are present in a diseased mathematical model consisting of 35 components, 32 reactions, and 62 kinetic laws (Figure 6A). Simulation graphs depict key regulatory components, namely, Atrogin1/Murf1, ERK, MEK, and I. FOXO1 (Figure 6B). PCA analysis depicted the important signaling molecules reducing the noise associated with STAT-3 signaling, NFκB signaling, and activation of FOXO-1 (Figure 6C). High flux reactions consisted of translocation of Atrogin1, Murf1, FOXO1, and NFκB and activation of FOXO1by SMAD2/3 complex (Figure 6D). Finally, quasipotential analysis of flux, concentration, and sensitivity produced a dome-shaped landscape (Figure 6E), reflecting the dynamic stability states underlying muscle atrophy in the cancer context. Crosstalk points observed for the model comprise Atrogin1, Murf1, CEBP-δ, NFκB, FOXO1, ActIIRB complex, and TAK1 (Figure 6F). Additional information regarding reaction and parameters are given in Supplementary Files S8, S9 and S11.

### 2.7. Cell Line Expression of miRNA

Quantitative real-time PCR (qPCR) analysis revealed the differential expression of miR-486-5p across various lung cancer and normal cell lines (Figure 4F). Relative expression levels were calculated using the 2^−ΔΔCt^ method, and normalization was done using the U6 internal control. The results reveal that miR-486-5p was significantly downregulated in the NSCLC samples (A549, H1975, and H1299) compared with the control (Beas-2b) (Supplementary File S12).

### 2.8. FOXO1 Expression in Co-Cultured C2C12 Using Confocal Imaging

Normal muscle cell line C2C12 were co-cultured with different lung cancer cell lines Beas-2b, H1299, and A549, with the result depicting a significant upregulation of FOXO-1 expression in cocultured cells (Figure 4G) when compared with only C2C12 cells. The obtained results indicate that the C2C12 cells came in to contact with the cancer cell media containing secretory molecules, which resulted in activation of pathways associated with FOXO1 synthesis, and thus a higher fluorescence intensity was measured in the case of C2C12 co-cultured with A549 and H1299 (Figure 4H) (Supplementary File S13).

Among the several differentially expressed miRNAs, hsa-miR-486-5p was found to target FOXO-1, which is a crucial regulator of autophagy, and further leads to activation of Atrogin1 and Murf1 E3 ubiquitin ligases, which leads to degradation of muscle proteins. Through our study, it was found that lower expression of hsa-miR-486-5p in cancer samples compared with normal samples indicates a critical role of curated miRNA in cancer progression, which needs to be explored. Further, expression of FOXO-1 in muscle cells increased with shared media from cancer cells, which directs towards an important interplay of FOXO-1 in both muscle and cancer cells. Our results pave the way for further mechanistic investigations on the role of microRNA and FOXO-1 in promoting NSCLC.

## 3. Discussion

NSCLC is one of the leading causes of death worldwide. Although therapies are available, they show limitations and resistance, leading to the new therapeutic approach of miRNA-based therapy [35]. These miRNAs can regulate different hallmarks of lung cancer. In this study, we used different miRNA sequencing datasets, namely, TCGA, GEO2R, CancerMIRNome, and small RNA sequencing data to identify a shared miRNA. Hsa-miR-486-5p was consistently downregulated across all datasets, suggesting a potential tumor-suppressive role in NSCLC. This finding aligns with previous studies reporting reduced miR-486-5p expression in lung cancer and its involvement in regulating cell proliferation. Our analysis revealed that hsa-miR-486-5p could be a crucial miRNA common between all the used datasets, highlighting the stringency of the cross-platform approach, along with the robustness of shared miRNAs, and whose significant downregulation suggests its role in lung cancer progression. Further, we performed a pathway enrichment study. The enrichment of pathways such as E2F targets, MYC signaling, and mTORC1 signaling indicates enhanced proliferative and metabolic activity, which are hallmarks of aggressive NSCLC. These findings are consistent with established oncogenic programs driven by KRAS and MYC, suggesting that the observed transcriptomic profile reflects a highly proliferative tumor state. In contrast, the negative enrichment of immune and differentiation-related pathways may indicate loss of tissue homeostasis and immune evasion, both of which are critical for tumor progression. This dual pattern provides an essential biological context for the interpretation of miRNA-mediated regulatory effects. Coordinated enrichment within the LUAD transcriptome was demonstrated by miRNA-centric GSEA using the 62 predicted target genes of hsa-miR-486-5p, indicating that these targets are not randomly distributed but instead function as a regulatory module. Several key genes involved in PI3K–AKT signaling, autophagy, and metabolic control—including FOXO1, PTEN, PIK3R1, IGF1, and CDK4—contribute significantly to this enrichment obtained by leading-edge analysis. The identification of FOXO1 as a high-confidence target of miR-486-5p provides a mechanistic link between miRNA dysregulation and key signaling pathways in NSCLC. While FOXO1 has traditionally been considered a tumor suppressor, emerging evidence suggests context-dependent roles, particularly in metabolic adaptation and stress responses [36]. Our findings support this dual role and indicate that FOXO1 may contribute to tumor survival under adverse conditions. By converging multiple prediction platforms, FOXO1 was highlighted as a biologically meaningful regulatory interaction providing a mechanistic basis for downstream analyses. Performing experimental assays, such as a luciferase reporter assay, would provide a more accurate understanding of the actual interaction and regulatory relationship between the identified miRNA–mRNA pairs.

Further to validating FOXO1 as a predicted target, we used a molecular interaction study involving molecular docking, with the minimum free energy of hsa-miR-486-5p-FOXO1 duplex confirming a strong binding affinity. Additionally, when this duplex docked with the Argonaute protein, it revealed a stable interaction with a highly negative binding free energy (ΔG) −56.7 kcal/mol.

**Figure 7:**
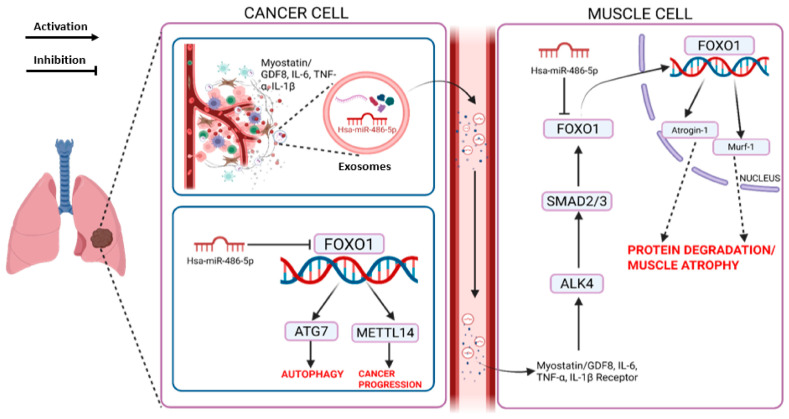
Schematic representation of microRNA-mediated regulation of FOXO1 and cytokine-driven tumor–muscle crosstalk contributing to protein degradation. The figure illustrates the dual mechanisms linking tumor progression to muscle wasting. Altered FOXO1 signaling influences downstream pathways involved in cell survival, metabolism, and stress responses.

We found FOXO1 to be an important target, involved in various signaling axes mediating autophagy and metabolism, ultimately leading to NSCLC progression. A comprehensive network of FOXO1-driven signaling was studied using a systems biology approach. Two mathematical models were reconstructed for cancer and muscle cells. The first model delineates oncogenic signaling cascades involving EGFR, AKT, SOS, hypoxia-driven pathways, and autophagy, converging on the regulatory role of FOXO1 in tumor progression. This framework illustrates how autophagy, activated by growth factor signaling and hypoxic adaptation, functions as a survival mechanism that enables tumor cells to recycle nutrients and sustain proliferation under metabolic stress. By linking upstream AKT signaling to downstream autophagic and metabolic processes, FOXO1 emerges as a pivotal transcriptional regulator orchestrating tumor resilience. Complementing this, the second model captures systemic consequences by focusing on skeletal muscle homeostasis. It demonstrates how tumor-derived inflammatory mediators activate catabolic signaling, thereby driving muscle wasting and contributing to cancer-associated sarcopenia. FOXO1 may serve as an integrative node connecting metabolism, muscle homeostasis, and oncogenic signaling, according to the consistent detection of this protein across several analytical techniques. FOXO1 is known to regulate autophagy and proteolysis processes essential for both tumor adaptability and cancer-associated muscle wasting, which is especially pertinent. These results point to a possible mechanistic link between the development of NSCLC and sarcopenia (Figure 7).

Corroborating in silico predictions and establishing relevance in biologically distinct NSCLC models, experimental validation using qRT-PCR confirmed significant downregulation of hsa-miR-486-5p in NSCLC cell lines H1299 and H1975. Later, a co-culture experiment consisting of C2C12 with NSCLC cell lines (NSCLC–sarcopenia model) confirmed that FOXO1-mediated signaling leads to NSCLC progression and sarcopenia. Taken together, this study suggests that the miR-486-5p–FOXO1 axis may represent a critical regulatory mechanism linking tumor progression with systemic metabolic alterations. The dual role of FOXO1 in promoting tumor cell survival while contributing to muscle protein degradation highlights its potential as a therapeutic target. However, given the context-dependent nature of FOXO1 activity, further experimental and clinical studies are required to validate its role in NSCLC-associated sarcopenia.

## 4. Methods

### 4.1. Differential Expression Analysis of microRNAs in NSCLC

#### 4.1.1. The Cancer Genome Atlas

miRNA expression datasets were retrieved for NSCLC through the GDC portal in TCGA (https://portal.gdc.cancer.gov/projects/tcga-luad Accessed: 12 October 2025), [37]. We have performed differential expression analysis with lung adenocarcinoma primary tumor and normal solid tissue using the GDC data portal and the TCGAbiolinks v2.36.0 package in R/Bioconductor v3.21. The filters applied were transcriptome profiling for data category, miRNA expression quantification for data type, and the workflow type selected was BCGSC miRNA profiling. Files were downloaded using the GDC data transfer tool and further processed with GDCprepare in R [38]. Raw data were extracted, and a computable matrix was made using the SummarizedExperiment v1.38.1 package in R for further analysis. TCGA miRNA expression data obtained from the GDC portal were normalized using the reads per million mapped miRNA (RPM) method, whereby raw counts were scaled relative to the total miRNA-mapped reads per sample and multiplied by 10^6^ to correct for sequencing depth differences. Using the DESeq2 package v1.48.1, the data were normalized, dispersion values were provided, and a negative binomial distribution was estimated for the given dataset to obtain the differentially expressed miRNAs. The Benjamini–Hochberg false discovery rate (FDR) method was used to calculate the adjusted *p*-values [39].

#### 4.1.2. GEO2R

An extensive literature survey on exosomal miRNA in lung cancer resulted in GSE137140, constituting miRNA expression in the serum (blood) samples of lung cancer patients before the operation compared with the control, and GSE68951, constituting miRNA profiles in the plasma samples of lung cancer patients compared with non-cancerous lung diseases. These two accession IDs were fed into GEO2R, which is a publicly available web-based tool through the Gene Expression Omnibus (GEO) database (https://www.ncbi.nlm.nih.gov/geo/ Accessed: 15 October 2025) in NCBI [40]. Based upon the experimental conditions, the samples were defined as the control group and the disease group. Further, a processed expression matrix was retrieved containing the normalized expression of both groups, and subsequent analysis was done using the linear models for microarray data (limma v3.64.3) package in R (Version 4.5.1). Aberrant expression of miRNAs (fold change ≥ 1 and *p*-value ≤ 0.05) between the two groups was assessed, and the resulting miRNAs were considered for downstream analyses [41,42,43].

#### 4.1.3. Small RNA Sequencing

The NEBNext Multiplex small RNA prep set was used to prepare a small RNA (SR) sequencing library of A549, H1975 (lung cancer cell line), and Beas-2b (normal lung epithelial cell). An amount of 1 μg of total RNA was used as a starting material, followed by 3′-SR ligation, hybridized reverse transcription primer, and 5′-SR adaptor ligation. Illumina Novoseq 6000 platform (Illumina, Inc., San Diego, CA, USA) with read length 50 nt and single-end sequence layout was used for sequencing [44]. The quality of raw reads was checked through the FastQC (v0.12.1) application, the adaptor was removed through cutadapt, and the read length was filtered to range from 15 to 36 nt. The mirDeep2 algorithm was used by taking the human genome, from which we get the mapping percentage of the sequenced filtered reads. Mapped reads help to identify known and novel miRNAs. For the identification of known miRNAs, human miRNA sequences were taken as a reference. The known and novel miRNAs of all the samples were used for the differential estimation using the edgeR package v4.6.3 in RStudio. The threshold applied for differential expression analysis was log_2_FC > ±1 and the FDR value ≤ 0.05, for the different pairwise combinations.

We have integrated three different platforms for the identification of differentially expressed miRNAs in NSCLC tissue, exosomes present in serum or plasma, and in-house lung cancer cell line sequencing data. These biologically different sources resolve inter-sample variability, lessen platform-specific bias, and increase confidence in miRNAs that are consistently dysregulated in circulation, tissue, and cell lines.

#### 4.1.4. CancerMIRNome

CancerMIRNome is a specialized and integrative database containing information about pan-cancer expression of different miRNAs. The database compares the normal and cancerous samples to obtain the expression profiles of miRNAs. This database has inbuilt tools which automatically filter miRNAs on the basis of log2 fold change, *p*-values, and FDR adjustments. GSE137140 and GSE68951 were used for survival and AUC analysis [45].

### 4.2. Gene Set Enrichment Analysis (GSEA)

Gene set enrichment analysis was performed to identify miRNA-regulated biological pathways in LUAD. mRNA differential expression data (LUAD tumor and normal) were retrieved from the GSE10072 dataset [46]. Based on a signed statistical metric incorporating both magnitude and significance of differential expression, genes were ranked, calculated as the sign of log fold change multiplied by the negative log10 of the *p*-value. To generate a ranked gene list, genes with an absolute log fold change greater than 1 and a *p*-value < 0.05 were retained. Predefined gene sets from the Molecular Signatures Database (MSigDB, v2025.1) were used, including Hallmark pathways and miRNA target gene sets derived from TargetScan (Release 8.0) [47]. To ensure consistency between expression data and gene sets, all analyses were performed using gene symbols. Pre-ranked GSEA was conducted in GSEA software (Version v3.0) [48]. Statistical significance was assessed using normalized enrichment scores (NES) and false discovery rate (FDR)-adjusted *p*-values. By obtaining the leading-edge genes from significantly enriched miRNA target gene sets to identify the subset of target genes driving enrichment, and then using these enriched genes, GSEA was performed to comprehend the pathway-level alterations mediated by hsa-miR-486-5p.

### 4.3. In-Silico Prediction of miRNA Target Genes

The curated miRNA from previous analysis was used to predict the target genes based on their sequence complementarity. A combination of sequence-based prediction tools was used, such as miRDB v6.0 (https://mirdb.org/ Accessed: 3 November 2025), TargetScan v8.0 (https://www.targetscan.org/vert_80/ Accessed: 3 November 2025), RNAhybrid v2.1.2 (http://bibiserv.techfak.uni-bielefeld.de/rnahybrid Accessed: 5 November 2025), and a Knuth–Morris–Pratt (KMP) algorithm for better prediction. MiRDB (Version 6.0) is a machine-learning-based prediction tool that was utilized to predict the miRNA–mRNA interaction. Targets were curated based upon the seed region pairing, which is a significant stretch of 2–8 nucleotides present at the 5′-end of miRNA, which is crucial for its interaction with the 3′-end of mRNA. A miRDB score was generated for each target [49]. TargetScan predicted targets based on conserved sequence complementarity with the 3′-untranslated region of mRNA. It resulted in 6-mer, 7-mer, and 8-mer conserved sites, along with poorly conserved and non-conserved binding sites for miRNA. Contextual information regarding the binding site was retrieved based on AU content, accessible sites, and space with poly-A tail. More conserved targets with 7-mer or 8-mer matches with more negative context++ score were selected [50,51]. The thermodynamic stability of miRNA–mRNA interaction was calculated using RNAhybrid. It was used to analyze the energetically favourable sites to assess the binding between miRNA and the 3′-UTR of mRNA. The algorithm required FASTA files, which were obtained from miRDB and NCBI GenBank for miRNA sequence and mRNA sequence, respectively. Based on this input sequence, RNAhybrid calculated the minimum free energy (MFE) values for interactions, which suggested optimal base pairing and lower energy of hybridization. The lower MFE value showed the stable interaction between miRNA and the predicted target gene, and was selected for further screening [52].

Furthermore, a Knuth–Morris–Pratt (KMP) algorithm, a Python-based string-match pattern search program was utilized to search sequences that are exact or nearly matching to seed regions of miRNAs. The KMP algorithm efficiently detects patterns in larger text without repeatedly comparing it for rapid identification. The algorithm utilizes the longest prefix or suffix array (LPS), which demonstrates the overlap between patterns to prevent redundant comparisons and avoid previously used sequences while matching [44]. Targets are predicted by integrating all four platforms, and targets common in at least three methods of prediction were selected to avoid false positive results. The use of four different methods of target prediction conferred more reliable interactions between miRNA and predicted gene based upon complementary sequences, conservation across species, thermal stability, and string-based matching. Two targets with a predominant role in NSCLC progression were considered for further studies.

### 4.4. Molecular Docking

MicroRNA and target gene sequences were accessed using TargetScan v8.0 (https://www.targetscan.org/vert_80/ Accessed: 12 January 2025), and secondary structure was generated using the RNAFold (Version 2.7.0) web server. Further, the dot-and-bracket structure of microRNA and target gene was uploaded in RNAComposer (Version 1.0) to obtain the tertiary structure [53,54]. The structure of Argonaute (PDB ID 3F73) protein was retrieved from the Protein Data Bank (PDB). After checking the quality of the structure in Molprobity software (Version 4.4), docking was done using GRAMM. Selection of the most stable model was done using PDBePISA [55,56,57].

### 4.5. Simulation and Mathematical Modeling of NSCLC and Related Signaling Molecules

#### 4.5.1. Reconstruction of Mathematical Model

A mathematical model was reconstructed to explicate the dual role of autophagy, the impact of the intricate signaling of the autophagy–FOXO1 axis, and intercellular communication through secretory cytokines leading to NSCLC-mediated muscle atrophy. The model was reconstructed using Simbiology toolbox in MATLAB v7.11.1.866, which enabled efficient simulation and analysis of a dynamic biological system, consisting of plasma membrane (PM), cytoplasm, and nucleus, comprising crosstalk between FOXO1 and autophagic proteins. SimBiology is a program-based tool that uses a format, the systems biology markup language (SBML), for identification and examination of the mechanisms behind the interactions between different biological system species under predetermined conditions. The model represents the biological system as a set of ordinary differential equations (ODEs), which is then solved by a Simbiology toolbox solver [58]. This quantitative modeling technique serves the objective to offer insights regarding the prevalent proteins playing a significant role in FOXO-1–autophagy interplay, resulting in induction of several other cancer-related pathways. To build, simulate, and analyze the mathematical model, a protocol was adapted from Khandibharad et al. [59].

The SimBiology user interface allows users to visually represent the biological system in the form of species, compartments, and reactions, which can be modified using the block diagram editor present in the toolbox. Cellular organelles are illustrated by the compartments, and reactant and product molecules are presented by species. For each biological reaction, a rate law is allocated. A law of mass action was assigned for elementary reactions such as ligand–receptor reactions; association–dissociation, where the reaction directly depends on the concentration of reaction; enzyme-catalyzed reaction, where the protein modification reaction Michaelis–Menten equation was used; and cooperative reactions, such as the binding of transcription factors to promoters, where Hill’s kinetics were used. An initial concentration of 10^3^–10^6^ was taken for the species, and units were assigned for each reaction. The solver type used for simulation was ODE15s Stiff/NDFs, and the simulation time was kept 100 s, resulting in a state vs. time graph for each component of the model. Analysis of mathematical models was performed based on sensitivity analysis, PCA, flux analysis, reduction of model and crosstalk points [60].

#### 4.5.2. Sensitivity Analysis

Sensitivity analysis was employed to study the impact on models with respect to variations in model entities (species, reaction, and parameters). This can be performed to substantiate already existing information or to validate a new hypothesis. Sensitivity analyses are classified into two types based on the techniques used for sensitivity calculation. The global sensitivity analysis (GSA) utilizes Monte Carlo simulations and depicts the effect of a representative component and its contribution to all other components, which in turn alters model behavior. The local sensitive analysis (LSA) used in this model is derivative-based, which only works with ODE solvers. Herein, the sensitivity was calculated in a time-dependent manner for each species, with time-dependent derivatives considered in light of their initial concentration and parameter values. The sensitivity was calculated using the suite of nonlinear and differential/algebraic equation solver (SUNDIALS). In SimBiology, the auxiliary differential equations for the sensitivities are combined with the basic ODE system to generate a model for the estimation of local sensitivities. The subsequent equations are parameter-specific derivatives of the initial equations [61].

#### 4.5.3. Principal Component Analysis

Principal component analysis (PCA) helps in the curation of principal components by assigning a specific PCA score to each component, thereby minimizing the background noise and reducing the dimensionality of the data. PCA implementation in MATLAB was done using the score-coefficient approach, where an m x n sensitivity matrix (a) was created based upon LSA performed in the previous analysis [62]. The score coefficients were calculated using the following function:

>> [score_coefficient] = princomp(a)(1)

The scores and coefficients were calculated based upon Eigen decomposition of the covariance matrix. The score-coefficient table obtained from MATLAB v7.11.1.866 was imported into SigmaPlot 15.0 for graphical visualization of the score coefficient vs. components plot [63].

#### 4.5.4. Flux Analysis

The quantitative evaluation of reaction rates or flow of molecules in a biological system using stoichiometric constraints and ODEs is referred to as the flux analysis. It demonstrates the rate of flow of molecules across a network under specific conditions. In this study, we used Complex Pathway Simulator (COPASI V4.11) to evaluate the rate of conversion of reactants to products. COPASI imports the file of reconstructed mathematical model from MATLAB, wherein it solves ODEs for each reaction until it reaches the steady state, and gives the instantaneous flux (concentration/second) at a constant time depending upon different kinetic parameters [64]. Reactions with higher flux rates were selected for further studies.

#### 4.5.5. Model Reduction

Model reduction was carried out using SigmaPlot 15.0, employing a systems biology-based regression approach to statistically simplify the mathematical framework. This process eliminated non-significant components while retaining those with a substantial impact on the reconstructed model. To evaluate system stability, a quasipotential curve integrating flux, sensitivity, and concentration (as previously calculated) was generated and visualized as a three-dimensional mesh landscape. The presence of distinct minima and maxima basins illustrates the dynamic transitions within the system, while the differently colored regions highlight phenotypic shifts from stable to unstable states [65].

#### 4.5.6. Identification of Crosstalk Point

A crosstalk point in a reconstructed mathematical model is depicted as the central species where multiple pathways converge, explaining the significant role of that component in the stability of the biological system. The number of interactions for each component of the model helps us identify key regulatory components in the model. The crosstalk point is calculated by subtracting the total degree of a node from the degree of the node for a particular pathway [65].

### 4.6. Cell Lines and Culture

For the culture of the H-1975 (Cat. No. CRL-5908), H-1299 (Cat. No. CRL-5803), and Beas-2b (Cat. No. CRL-9609) cell lines, Rosewell Park Memorial Institute 1640 (RPMI-1640) was used. A549 cells were cultured in Ham’s F-10 (HF-10) media. All media were supplemented with 10% FBS, and penicillin–streptomycin solution, and cells were maintained at 37 °C with 5% CO_2_ in a humidified incubator.

### 4.7. RNA Isolation and cDNA Preparation

Cells were treated with 500 μL TRIzol (Invitrogen, Thermo Fisher Scientific, Waltham, MA, USA) reagent and collected. An amount of 100 μL of chloroform was added to each of the tubes followed by gentle mixing, and then incubated at room temperature (RT) for 15 min. Samples were then centrifuged for 15 min at 12,000 RPM at 4 °C. The top aqueous layer was collected and transferred to a different tube, 500 μL isopropanol was added to it, gently mixed by inverting, incubated for 10 min, and centrifuged at 12,000 RPM at 4 °C for 15 min. Pellets were washed with 75% of ethanol twice, after washing pellets were dried and reconstituted using 30 μL of diethylpyrocarbonate (DEPC) water. For quantification and analysis of RNA extraction, a Thermo Scientific Multiskan SkyHigh multispectrophotometer (Thermo Fisher Scientific, Waltham, MA, USA) was used to record absorbance at 280/260 and 260/230 ratios. cDNA preparation was done using 10 μL of 100 ng/mL cell suspension mixed with reagents from the cDNA synthesis kit (Thermofisher Scientific ^TM^). To this mixture, 3 μL of hsa-miR-486-5p primer (4427975 Thermofisher Scientific) was added, and another tube for U6 internal control was made similarly. The samples were mixed thoroughly by centrifugation and loaded onto a thermal cycler for the cyclic reaction.

### 4.8. Q-PCR

Differential miRNA expression analysis was performed using a 100 ng/μL concentration of cDNA from each sample. A total of 10 μL of the reaction mixture was utilized for analysis, consisting of (2X) TaqMan Fast Universal PCR Master Mix (4366072 Thermofisher Scientific), cDNA samples, and 0.5 μL of hsa-miR-486-5p probes (4427975 Thermofisher Scientific) along with nuclease-free water to make up the entire volume. To perform the qRT-PCR, the StepOne plus Real Time PCR machine (Thermo Fisher Scientific, Waltham, MA, USA) and StepOne software version 2.3 were used. The PCR cycle followed for analysis comprised steps holding at 50 °C for 2 min, holding at 95 °C for 20 s, cycling at 95 °C for 3 s, and cycling at 60 °C for 30 s. There were forty cycles in total, and comparative CT (ΔΔ CT) was recorded for each sample. To compare each sample, a U6 internal control probe was used.

### 4.9. Co-Culture of Cancer and Muscle Cells

Six-well co-culture plates were seeded with 10^5^ cells of the C2C12 cell line and inserts of pore size 0.4 µM containing 10^6^ cells of the cell lines Beas-2b, A549, and H1299 were cultured for 24 h, separately. After proper adherence, inserts containing cancer cell lines were kept on the wells of the C2C12 muscle cell line for 48 h, such that a shared medium is present for the transfer of small molecules between them.

### 4.10. Confocal Microscopy

After 48 h, the co-cultured cells were seeded in a 96-well plate with 10^4^ cells seeding density and allowed to incubate for 24 h at 37 °C and 5% CO_2_. Incubation was followed by three washes using 1X PBS and 0.05% Tween-20 (3601181001730 Genei, Karnataka, India), further fixation was done using 4% para-formaldehyde for 10 min. After washing with 1X PBS, cells were incubated for 10 min in 1X PBST. Blocking was done with 3% bovine serum albumin (BSA) for 30 min at RT. Cells were washed with 1X PBST, and FOXO-1 primary antibody (1:1000) dilution was added and incubated for 2 h at RT. After washing, cells were treated with mouse phalloidin (Alexa Fluor 568) anti-rabbit IgG (H+L) F (ab’)2 fragments (Alexa Fluor 488 conjugate) at a dilution of 1:500 for both and incubated for an hour. Cells were washed three times with 1X PBST before counterstaining with DAPI for 10 min at RT. Cells were then washed with 1X PBST three times. Confocal imaging was performed using an Olympus FV3000 confocal microscope (Olympus Corporation, Tokyo, Japan) at 60X magnification, and processing of acquired images was done using Fiji version 1.54k.

### 4.11. Statistical Analysis

All the quantitative data were derived from at least two independent experiments showing consistent outcomes. The data were represented as mean ± standard deviation (SD). For statistical analysis, one-way ANOVA was used followed by Dunnett’s multiple comparisons test to evaluate variations across the groups. Asterisks represent statistical significance as follows: *p* < 0.05 (*), *p* < 0.01 (**), *p* < 0.001 (***) and *p* < 0.0001 (****). GraphPad Prism 5 for Windows (Graphpad Software, La Jolla, CA, USA) was used to carry out the analysis.

## 5. Challenges and Future Perspectives

Given that most miRNA-based therapies are still in preliminary or early phases of human clinical trials, it remains to be determined how these treatments perform with respect to toxicity and adverse effects. To further validate the role of miRNAs in muscle atrophy, additional studies are needed to examine common miRNAs in samples from patients with sarcopenia. A major concern in miRNA therapeutics lies in their specificity and sensitivity. Because a single miRNA can regulate multiple biological pathways by targeting several genes and that, conversely, many miRNAs can act on the same mRNA, this complexity makes miRNAs powerful therapeutic molecules but also poses challenges in minimizing off-target effects observed in clinical studies. Looking ahead, targeted delivery strategies such as extracellular vesicle packaging, lipid-based carriers, and polymer nanoparticles hold promise for enhancing the therapeutic efficacy of miRNA treatments while reducing potential adverse outcomes.

## 6. Conclusions

In conclusion, our study demonstrates that hsa-miR-486-5p is downregulated in NSCLC and directly targets FOXO1-mediated signaling. Insights from signaling models highlight FOXO1 as a critical mediator of crosstalk between NSCLC and muscle cells. Consequently, targeting FOXO1 through hsa-miR-486-5p may represent a promising therapeutic strategy for addressing NSCLC-associated sarcopenia, offering new avenues for intervention in both tumor progression and muscle wasting.

## Data Availability

The original contributions presented in this study are included in the article/Supplementary Material. Further inquiries can be directed to the corresponding author.

## Supplementary Materials

The following supporting information can be downloaded at: https://www.mdpi.com/article/10.3390/ijms27114703/s1.

## Abbreviations

The following abbreviations are used in this manuscript:

AKT	Protein kinase B
ALK	Anaplastic lymphoma kinase
AMPK	AMP-activated protein kinase
ASO	Antisense oligonucleotide
AUC	Area under the curve
CTLA-4	Cytotoxic T-lymphocyte-associated protein 4
DEPC	Diethyl pyrocarbonate
DROSHA	RNase III endonuclease drosha
EGFR	Epidermal growth factor receptor
EMT	Epithelial-to-mesenchymal transition
FOXO	Forkhead box O
FOXO1	Forkhead box O1
GDC	Genomic data commons
GEO	Gene expression omnibus
GSEA	Gene set enrichment analysis
ICI	Immune checkpoint inhibitor
ICMR	Indian Council of Medical Research
IL-1β	Interleukin-1 beta
IL-6	Interleukin-6
JAK	Janus kinase
JNK	c-Jun N-terminal kinase
KMP	Knuth–Morris–Pratt
KRAS	Kirsten rat sarcoma
LASSO	Least absolute shrinkage and selection operator
LDL	Low-density lipoprotein
LPS	Lipopolysaccharide
LUAD	Lung adenocarcinoma
miRNAs	microRNAs
mTOR	Mechanistic target of rapamycin
NCDIR	National Centre for Disease Informatics and Research
NCRP	National Cancer Registry Programme
NES	Normalized enrichment score
NF-κB	Nuclear factor kappa-light-chain-enhancer of activated B cells
NSCLC	Non-small cell lung cancer
ODE	Ordinary differential equation
OS	Overall survival
PCA	Principal component analysis
PD-1	Programmed cell death protein 1
PDB	Protein Data Bank
PDCD4	Programmed cell death protein 4
PD-L1	Programmed death ligand 1
PI3K	Phosphoinositide 3 kinase
PTEN	Phosphatase and tensin homolog
RISC	RNA-induced silencing complex
ROC	Receiver operating characteristic
ROS	Reactive oxygen species
SAPK	Stress-activated protein kinase
SBML	Systems biology markup language
SCLC	Small cell lung cancer
siRNAs	Small interfering RNAs
SMN	Survival of motor neuron
SMN2	Survival of motor neuron 2
STAT3	Signal transducer and activator of transcription 3
TAM	Tumor-associated macrophage
TCGA	The Cancer Genome Atlas
TKI	Tyrosine kinase inhibitor
TME	Tumor microenvironment
TNF-α	Tumor necrosis factor alpha
TPM1	Tropomyosin 1
ULK2	Unc-51-like autophagy activating kinase 2
WHO	World Health Organization

## References

[1] Sharma R., Khubchandani J. (2024). Global, regional, and national burden of tracheal, bronchus, and lung cancer in 2022: Evidence from the GLOBOCAN study. Epidemiologia.

[2] Nath A., Sathishkumar K., Das P., Sudarshan K.L., Mathur P. (2022). Clinicoepidemiological profile of lung cancers in India: Results from the National Cancer Registry Programme. Indian J. Med. Res..

[3] Sathishkumar K., Chaturvedi M., Das P., Stephen S., Mathur P. (2022). Cancer incidence estimates for 2022 and projection for 2025: Results from the National Cancer Registry Programme, India. Indian J. Med. Res..

[4] Zhang Y., Vaccarella S., Morgan E., Li M., Etxeberria J., Chokunonga E., Manraj S.S., Kamate B., Omonisi A., Bray F. (2023). Global variations in lung cancer incidence by histological subtype in 2020. Lancet Oncol..

[5] Rudin C.M., Ismaila N., Hann C.L., Malhotra N., Movsas B., Norris K., Johnson D.H. (2015). Treatment of small-cell lung cancer: ASCO endorsement of the ACCP guideline. J. Clin. Oncol..

[6] Clark S.B., Alsubait S. (2023). Non–Small Cell Lung Cancer [Updated 4 September 2023]. StatPearls [Internet].

[7] Mak K.S., Gainor J.F., Niemierko A., Oh K.S., Willers H., Choi N.C., Loeffler J.S., Sequist L.V., Shaw A.T., Shih H.A. (2015). Significance of targeted therapy and genetic alterations in EGFR, ALK, or KRAS on survival in non-small cell lung cancer patients with brain metastases. Neuro-Oncology.

[8] Scagliotti G.V., Parikh P., von Pawel J., Biesma B., Vansteenkiste J., Serwatowski C.M., Serwatowski P., Gatzemeier U., Digumarti R., Zukin M. (2008). Phase III study comparing cisplatin plus gemcitabine with cisplatin plus pemetrexed in chemotherapy-naive patients with advanced-stage non-small-cell lung cancer. J. Clin. Oncol..

[9] Jeon H., Wang S., Song J., Gill H., Cheng H. (2025). Update 2025: Management of Non-Small-Cell Lung Cancer. Lung.

[10] Smolarz B., Łukasiewicz H., Samulak D., Piekarska E., Kołaciński R., Romanowicz H. (2025). Lung Cancer—Epidemiology, Pathogenesis, Treatment and Molecular Aspect (Review of Literature). Int. J. Mol. Sci..

[11] Tufail M., Jiang C.H., Li N. (2025). Tumor dormancy and relapse: Understanding the molecular mechanisms of cancer recurrence. Mil. Med. Res..

[12] Uramoto H., Tanaka F. (2014). Recurrence after surgery in patients with NSCLC. Transl. Lung Cancer Res..

[13] Bartel D.P. (2004). MicroRNAs: Genomics, biogenesis, mechanism, and function. Cell.

[14] Rupaimoole R., Slack F.J. (2017). MicroRNA therapeutics: Towards a new era for the management of cancer and other diseases. Nat. Rev. Drug Discov..

[15] Zhu Y., Zhu L., Wang X., Jin H., Li Y. (2022). RNA-based therapeutics: An overview and prospectus. Cell Death Dis..

[16] Zhang X., Liu C., Li H., Guo L. (2020). Effects of miR-21 on proliferation and apoptosis of WT cells via the PTEN/Akt pathway. Exp. Ther. Med..

[17] Di Martino M.T., Arbitrio M., Caracciolo D., Cordua A., Cuomo O., Grillone K., Riillo C., Caridà G., Scionti F., Labanca C. (2022). miR-221/222 as biomarkers and targets for therapeutic intervention in cancer and other diseases: A systematic review. Mol. Ther. Nucleic Acids.

[18] Di Martino M.T., Tagliaferri P., Tassone P. (2025). MicroRNA in cancer therapy: Breakthroughs and challenges in early clinical applications. J. Exp. Clin. Cancer Res..

[19] Wu J., Zhu Y., Liu D., Cong Q., Bai C. (2024). Biological functions and potential mechanisms of miR-143-3p in cancers. Oncol. Rep..

[20] Mozammel N., Amini M., Baradaran B., Mahdavi S.Z.B., Hosseini S.S., Mokhtarzadeh A. (2023). The function of miR-145 in colorectal cancer progression: An updated review on related signaling pathways. Pathol.—Res. Pract..

[21] Tan S., Xia L., Yi P., Han Y., Tang L., Pan Q., Liu J. (2020). Exosomal microRNAs in the tumor microenvironment. J. Exp. Clin. Cancer Res..

[22] Santos P., Almeida F. (2020). Role of exosomal microRNAs and the tumor microenvironment in drug resistance. Cells.

[23] He Y., Sun M.M., Zhang G.G., Yang J., Chen K.S., Xu W.W., Li B. (2021). Targeting PI3K/Akt signal transduction for cancer therapy. Signal Transduct. Target. Ther..

[24] Gao Z.J., Yuan W.D., Yuan J.Q., Yuan K., Wang Y. (2018). miR-486-5p functions as an oncogene by targeting PTEN in non-small cell lung cancer. Pathol. Res. Pract..

[25] Chen Z., Han F., Du Y., Shi H., Zhou W. (2023). Hypoxic microenvironment in cancer: Molecular mechanisms and therapeutic interventions. Signal Transduct. Target. Ther..

[26] Pomiès P., Blaquière M., Maury J., Mercier J., Gouzi F., Hayot M. (2016). Involvement of the FoxO1/MuRF1/Atrogin-1 signaling pathway in the oxidative stress-induced atrophy of cultured chronic obstructive pulmonary disease myotubes. PLoS ONE.

[27] Ma J.F., Sanchez B.J., Hall D.T., Tremblay A.K., Di Marco S., Gallouzi I.E. (2017). STAT3 promotes IFNγ/TNFα-induced muscle wasting in an NF-κB-dependent and IL-6-independent manner. EMBO Mol. Med..

[28] Sandri M., Sandri C., Gilbert A., Skurk C., Calabria E., Picard A., Walsh K., Schiaffino S., Lecker S.H., Goldberg A.L. (2004). FoxO transcription factors induce the atrophy-related ubiquitin ligase atrogin-1 and cause skeletal muscle atrophy. Cell.

[29] Fanzani A., Conraads V.M., Penna F., Martinet W. (2018). Molecular and cellular mechanisms of skeletal muscle atrophy: An update. J. Muscle Res. Cell Motil..

[30] Jeong J.Y., Son Y., Kim Y., Lee N., Kim Y., Heo Y.J., Choi S.E., Choi J., Han S.J., Jeon J. (2025). Deferoxamine prevents dexamethasone-induced muscle atrophy by reducing MuRF1 and atrogin-1. Front. Pharmacol..

[31] Zhou X., Wang J.L., Lu J., Song Y., Kwak K.S., Jiao Q., Rosenfeld R., Chen Q., Boone T., Simonet W.S. (2010). Reversal of cancer cachexia and muscle wasting by ActRIIB antagonism leads to prolonged survival. Cell.

[32] Zhang P., Cheng S., Sheng X., Dai H., He K., Du Y. (2021). The role of autophagy in regulating metabolism in the tumor microenvironment. Genes Dis..

[33] Folkerts H., Hilgendorf S., Vellenga E., Bremer E., Wiersma V.R. (2019). The multifaceted role of autophagy in cancer and the microenvironment. Med. Res. Rev..

[34] Liberzon A., Subramanian A., Pinchback R., Thorvaldsdóttir H., Tamayo P., Mesirov J.P. (2011). Molecular signatures database (MSigDB) 3.0. Bioinformatics.

[35] Ding L., Tian W., Zhang H., Li W., Ji C., Wang Y., Li Y. (2021). MicroRNA-486-5p suppresses lung cancer via downregulating mTOR signaling in vitro and in vivo. Front. Oncol..

[36] Chai S., Li X., Ding F., Wang X., Xu J. (2019). miR-486-5p inhibits inflammatory response, matrix degradation, and apoptosis by directly targeting FOXO1. Cell. Physiol. Biochem..

[37] National Cancer Institute, National Human Genome Research Institute (2006). The Cancer Genome Atlas (TCGA).

[38] Hu Y., Dingerdissen H., Gupta S., Kahsay R., Shanker V., Wan Q., Yan C., Mazumder R. (2018). Identification of key differentially expressed microRNAs in cancer patients through pan-cancer analysis. Comput. Biol. Med..

[39] Moradi S., Kamal A., Aboulkheyr Es H., Farhadi F., Ebrahimi M., Chitsaz H., Sharifi-Zarchi A., Baharvand H. (2022). Pan-cancer analysis of microRNA expression profiles highlights microRNAs enriched in normal body cells as effective suppressors of multiple tumor types: A study based on TCGA database. PLoS ONE.

[40] National Center for Biotechnology Information (2012). GEO2R: Analyze GEO Data [Video]. https://digitalcommons.imsa.edu/bioinfo_gene/1.

[41] Asakura K., Kadota T., Matsuzaki J., Yoshida Y., Yamamoto Y., Nakagawa K., Takizawa S., Aoki Y., Nakamura E., Miura J. (2020). A miRNA-based diagnostic model predicts resectable lung cancer in humans with high accuracy. Commun. Biol..

[42] Xia Y., Wei K., Yang F.M., Hu L.Q., Pan C.F., Pan X.L., Wu W.B., Wang J., Wen W., He Z.C. (2019). miR-1260b, mediated by YY1, activates KIT signaling by targeting SOCS6 to regulate cell proliferation and apoptosis in NSCLC. Cell Death Dis..

[43] Ritchie M.E., Phipson B., Wu D., Hu Y., Law C.W., Shi W., Smyth G.K. (2015). Limma powers differential expression analyses for RNA-sequencing and microarray studies. Nucleic Acids Res..

[44] Gautreau I. Protocol for Use with NEBNext^®^ Small RNA Library Prep Set for Illumina^®^. Protocols.io. 2018 ManualE7300 Page no.6. https://www.protocols.io/view/protocol-for-use-with-nebnext-small-rna-library-pr-yxmvm7n9bv3p/v1.

[45] Li R., Qu H., Wang S., Chater J.M., Wang X., Cui Y., Yu L., Zhou R., Jia Q., Traband R. (2022). CancerMIRNome: An interactive analysis and visualization database for miRNome profiles of human cancer. Nucleic Acids Res..

[46] Zhou J., Mu M., Xing Y., Zhang X., Li D., Liu Y., Xie J., Hu W., Zhang L., Wu J. (2020). Identification of Key Genes in Lung Adenocarcinoma and Establishment of Prognostic Mode. Front. Mol. Biosci..

[47] Wang Y., Hong Y., Mao S., Jiang Y., Cui Y., Pan J., Luo Y. (2022). An Interaction-Based Method for Refining Results From Gene Set Enrichment Analysis. Front. Genet..

[48] Reimand J., Isserlin R., Voisin V., Kucera M., Tannus-Lopes C., Rostamianfar A., Wadi L., Meyer M., Wong J., Xu C. (2019). Pathway enrichment analysis and visualization of omics data using g:Profiler, GSEA, Cytoscape and EnrichmentMap. Nat. Protoc..

[49] Wang X. (2019). miRDB: An online database for prediction of functional microRNA targets. Nucleic Acids Res..

[50] Lewis B.P., Burge C.B., Bartel D.P. (2005). Conserved Seed Pairing, Often Flanked by Adenosines, Indicates that Thousands of Human Genes are MicroRNA Targets. Cell.

[51] Grimson A., Farh K.K.H., Johnston W.K., Garrett-Engele P., Lim L.P., Bartel D.P. (2007). MicroRNA targeting specificity in mammals: Determinants beyond seed pairing. Mol. Cell.

[52] Rehmsmeier M., Steffen P., Höchsmann M., Giegerich R. (2004). Fast and effective prediction of microRNA/target duplexes. RNA.

[53] Popenda M., Szachniuk M., Antczak M., Purzycka K.J., Łukasik P., Bartol N., Błazewicz J., Adamiak R.W. (2012). Automated 3D structure composition for large RNAs. Nucleic Acids Res..

[54] Wang Y., Juranek S., Li H., Sheng G., Wardle G.S., Tuschl T., Patel D.J. (2009). Structure of an Argonaute silencing complex with a seed-containing guide DNA and target RNA duplex. Nature.

[55] Tovchigrechko A., Vakser I.A. (2006). GRAMM-X public web server for protein–protein docking. Nucleic Acids Res..

[56] Krissinel E., Henrick K. (2007). Inference of macromolecular assemblies from crystalline state. J. Mol. Biol..

[57] Vakser I.A. (2014). Protein–protein docking: From interaction to interactome. Biophys. J..

[58] MathWorks (2011). SimBiology^®^ User’s Guide.

[59] Khandibharad S., Gulhane P., Singh S. (2025). Computational cellular mathematical model aids understanding the cGAS–STING pathway in NSCLC pathogenicity. Bio-Protocol.

[60] Rani D., Khandibharad S., Singh S. (2025). Integrative modeling of FOXO-mediated autophagy in NSCLC: Linking cGAS–STING signaling to IL-6 dynamics. Front. Oncol..

[61] Marino S., Hogue I.B., Ray C.J., Kirschner D.E. (2008). A methodology for performing global uncertainty and sensitivity analysis in systems biology. J. Theor. Biol..

[62] MathWorks (2010). Statistics and Machine Learning Toolbox: Principal Component Analysis (PCA) in MATLAB.

[63] Systat Software, Inc (2021). SigmaPlot.

[64] Hoops S., Sahle S., Gauges R., Lee C., Pahle J., Simus N., Kummer U. (2022). COPASI.

[65] Kumar G., Khandibharad S., Singh S. (2025). Targeting IL-6/STAT3 signaling to mitigate sarcopenia: Insights from immuno-metabolic crosstalk in NSCLC. Biochem. Biophys. Res. Commun..

